# The Impact of COVID-19-Related Psychological Stress on Fetoplacental Circulation: A Doppler Scan of Umbilical Vessels in Third-Trimester Antenatal Women

**DOI:** 10.7759/cureus.41517

**Published:** 2023-07-07

**Authors:** M Gowtham, T.S. Gugapriya, Ashwini A Umredkar, Snehal Deulkar

**Affiliations:** 1 Anatomy, All India Institute of Medical Sciences Nagpur, Nagpur, IND; 2 Radio-diagnosis, All India Institute of Medical Sciences Nagpur, Nagpur, IND

**Keywords:** preps tool, psychological stress, pulsatility index, umbilical vein blood flow, pregnant women, umbilical vein, pandemic, covid-19

## Abstract

Introduction: The placenta is an important organ of pregnancy. A multitude of physiological and pathological factors influence blood flow in the placenta during pregnancy. However, the fetal effects of maternal psychological stress were inconclusive. The recent COVID-19 pandemic had unprecedented economic, social, and psychological effects. The effect of COVID-19-induced psychological stress in antenatal women and its resultant fetal impact were studied by observing the Doppler waveforms of the uterine and umbilical arteries.

Methods: The cross-sectional study was conducted on 26 healthy third-trimester antenatal women who satisfied pre-set inclusion and exclusion criteria. A pandemic-related pregnancy stress scale (PREPS) was used to evaluate the stress in pregnant women and categorize it into mild, moderate, and severe levels. The Doppler ultrasound of the uterine and umbilical vessels was done along with a routine growth scan in the third trimester of pregnancy. The arterial waveforms, Pulsatility index (PI) of uterine and umbilical arteries, umbilical vein blood flow, and biometric parameters of the fetus were recorded and analyzed.

Results: Seventeen of the 26 participants were found to be moderately stressed. Among the three dimensions of the PREPS tool, the perinatal infection stress dimension was expressed predominantly. A strong expression of the positive affirmation dimension was seen in antenatal women. The mean Pulsatility index in the mild, moderate, and severe groups was 0.74, 0.93, and 0.63, respectively. The association between the PREPS score and the Pulsatility index of the umbilical artery alone was found to be significant at p=0.02.

Conclusion: The COVID-19 pandemic caused moderate to severe levels of psychological stress in pregnant women. The statistically significant association between the PREPS score and the umbilical artery PI indicates possible fetoplacental compromise, suggesting the need for cognitive therapy to manage psychological stress in antenatal women.

## Introduction

The placenta, a distinctive organ of pregnancy, is made up of two separate vascular compartments, namely the uteroplacental and fetoplacental circulations [1]. Continuous structural remodeling happens in the trophoblastic villi of the fetoplacental compartment throughout pregnancy [2]. In comparison, the vascular maturation in the uteroplacental compartment is completed by the early second trimester of gestation [3]. Ultrasonography and Doppler flow measurements of uterine and umbilical vessels provide means to visualize placental and fetal growth and development during gestation [3].

Apart from maternal body mass index (BMI) and age, certain physiological states like rest, sleep, food intake, and activity positively affect the umbilical blood flow [3,4]. The physical as well as psychological status of the mother had been believed to impact the unborn child. Literature evidence claims that psychological stress exerts a negative influence not only on pregnancy outcomes but also on the physiological and psychological development of the offspring [1,5,6]. Inconsistent relations between maternal psychological stress and an immediate adverse fetal outcome like fetal growth retardation, small for gestational age, preterm labor, and reduced birth weight have been observed [5,7,8].

The current COVID-19 pandemic has been confirmed to change lives, daily routines, work, social life, and family life, resulting in unprecedented psychological stress and anxiety for pregnant women [9,10]. Several studies conducted in different countries suggest that the prevalence of anxiety and depression symptoms may have increased in pregnant women during the pandemic [11]. High rates of perceived stress, depression, and anxiety symptoms in pregnant COVID-19 patients were reported in India too [12].

The psychological stress response causes resistance to blood flow in the maternal uterine and umbilical arteries by activating the sympathetic adrenal medullary axis, thereby causing compromised fetoplacental circulation [13,14]. The pandemic-related pregnancy stress scale (PREPS), a multidimensional questionnaire, systematically evaluates psychological stress in pregnant women [15]. A study of umbilical vein blood flow in the third trimester showed a decrease in fetoplacental blood flow as the result of maternal stress [7].

Despite extensive research about the association between maternal psychological stress and Doppler findings indicative of fetoplacental compromise, there exists a knowledge gap that prevents arriving at a definitive conclusion because of substantial methodological differences across studies [16]. The recent COVID-19 pandemic documented the occurrence of maternal psychological stress [9-12]. And so, it is imperative to explore the impact of the unprecedented COVID-19 pandemic-induced psychological stress upon vulnerable pregnant women and the fetoplacental blood flow by Doppler ultrasonography in third-trimester pregnant women.

Hence, this cross-sectional analytical study was done to fill the lacunae in knowledge and data about the COVID-19 pandemic-induced psychological stress upon vulnerable pregnant women in the setting of the central India region.

## Materials and methods

Sample characteristics

All the antenatal women who reported to the Department of Radio-diagnosis for a third-trimester growth scan from July 2022 to August 2022 were considered sample populations for this cross-sectional clinical investigation. The study was conducted after obtaining an Institutional Ethical Committee clearance certificate (IEC/PHARMAC/2022/426, dated 22-06-22). The antenatal women aged between 20 and 35 years with singleton pregnancy and having a history of being infected with COVID-19 during the pregnancy period or family members affected by COVID-19 were included in the study sample after obtaining written informed consent. Those antenatal women with extremes of age, gestational comorbid states like gestational diabetes mellitus, eclampsia, and anemia, or who were already diagnosed as having high-risk pregnancies with multiple pregnancies, were excluded from this study.

Study tool

The pre-validated English version of the PREPS questionnaire consisted of 15 items distributed into three dimensions, namely PREPS preparedness stress, PREPS perinatal stress, and PREPS positive affirmation, containing seven, five, and three items, respectively [15]. It was translated and linguistically validated into the regional Language. According to the literacy level of the antenatal women, either the English or the regional version of the PREPS tool was used. The scoring was done on a 5-point scale, with 1 = very little to 5 = very much. The overall score ranges between 15 and 75. Based on the score obtained the participants were categorized into mild - 15-30; moderate - 31-60; severe - 61-75 categories. The dimension-wise analysis of the score was done by taking the cut-off scores for preparedness stress, perinatal infection stress, and positive affirmation as ≥21, ≥15, and ≥9, respectively.

Study method

The study method consists of two sequential steps.

Step I

The basic obstetric and demographic case histories were recorded. The PREPS questionnaire was administered. The Scoring of PREPS was calculated and graded as mild, moderate, or severe.

Step II

The study participants were subjected to a Doppler ultrasound of the uterine and umbilical vessels and a routine growth scan for the third trimester in a GE ultrasound machine, the LOGIQ-P9, with 3D and 4D color Doppler. The radiologist was blinded to the PREPS score to eliminate observer bias. The arterial waveforms in the uterine and umbilical arteries were recorded. The sample gate for the uterine artery was within 1 cm ventrally of the crossover with the external iliac artery. The umbilical artery was assessed in a free loop of the umbilical cord during fetal quiescence. Blood flow waveforms in the umbilical vein and the intraabdominal part of the vessels were done. The pulsatility index (PI) of uterine and umbilical vessels and maternal and fetal heart rates were measured for three waveforms. The difference between the peak systolic flow and minimum diastolic flow velocity is divided by the mean velocity recorded throughout the cardiac cycle in a non-invasive method using Doppler ultrasonography and is called the pulsatility index [17]. Growth parameters such as biparietal diameter, abdominal circumference, femur length, estimated fetal weight, and placental morphology were also recorded as part of a routine third-trimester scan.

Data analysis

Descriptive statistics were done using SPSS. Shapiro-Wilk W test for normality of data was performed. The measure of association between the PREPS scoring (independent variable) and the altered observation in Doppler ultrasound (dependent variable) was analyzed by ANOVA and the non-parametric Kruskal-Wallis test.

## Results

Demographic details

Among the study sample of 26, 22 antenatal women were in the young category of ≤30 years of chronological age. Twelve primi and eight low multipara gravida were found. Ninety-two percent of the participants were either not working or working as non-essential workers during the COVID-19 pandemic.

COVID-19 history

An inquiry into the history of COVID-19 infection showed that 15% had a history of being infected themselves, and 35% had a history of family members getting infected with COVID-19.

Impact of COVID-19

Household income and employment were affected by the COVID-19 pandemic for nearly 85% of the participants in this study. Despite COVID-19, 88% did not miss their antenatal checkup.

PREPS score

As per the PREPS score, 27%, 65%, and 8% of the study participants were in the mild, moderate, and severe categories of psychological stress induced by the pandemic, respectively. The dimension-wise analysis of the PREPS score showed perinatal infection stress was the predominant stress, followed by preparedness stress. The maximum number of participants who showed strong positive affirmation was seen as moderately stressed in the PREPS score category (Table 1).

**Table 1:** Shows different categories of PREPS scoring exhibited by antenatal women due to COVID-19-induced psychological stress PREPS: pandemic-related pregnancy stress scale.

PREPS score category	Number of participants who showed		
	Preparedness stress (score ≥21)	Perinatal infection stress (score ≥15)	Positive affirmation strongly (score ≥ 9)
Mild	0	0	7
Moderate	1	2	17
Severe	2	2	2

Doppler ultrasonography

**Figure 1:**
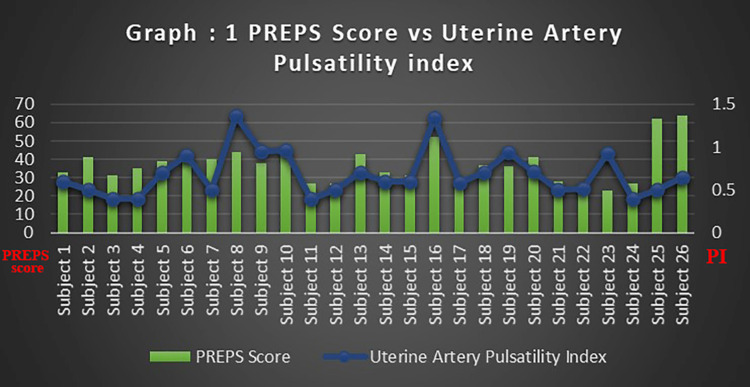
PREPS score versus uterine artery pulsatility index PREPS: pandemic-related pregnancy stress scale.

**Figure 2:**
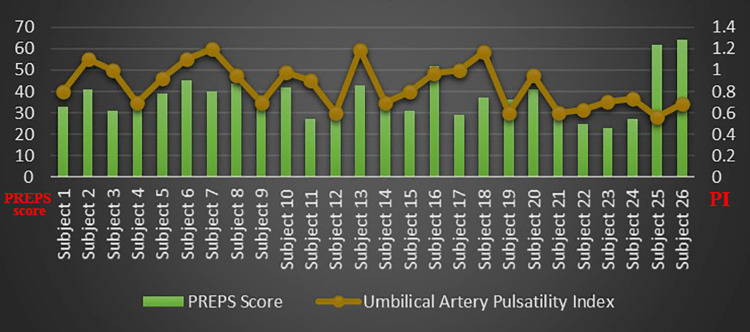
PREPS score versus umbilical artery pulsatility index PREPS: pandemic-related pregnancy stress scale.

**Figure 3:**
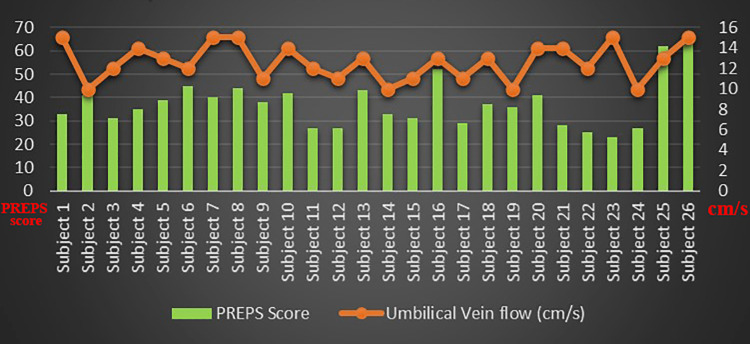
PREPS score versus umbilical vein flow PREPS: pandemic-related pregnancy stress scale.

The arterial waveforms of the uterine and umbilical arteries recorded were observed to be within normal limits for all the categories of the PREPS score. The PREPS score was correlated with the uterine artery, umbilical artery pulsatility index, and umbilical vein flow rate (Figures 1-3). The mean PI of the uterine artery in mild, moderate, and severe groups was 0.55, 0.76, and 0.57, respectively. The Shapiro-Wilk W test for the normality of data showed the uterine artery PI to slightly violate the normality assumption. Despite this, ANOVA was used for analysis considering its robustness. The association between the PREPS score and the PI of the uterine artery tested with ANOVA was found to be not significant at p=0.18. The mean PI of the umbilical artery in mild, moderate, and severe groups was 0.74, 0.93, and 0.63, respectively. The association between the PREPS score and the PI of the umbilical artery tested with ANOVA was found to be significant at p=0.02 (Table 2). The mean blood flow in the umbilical vein in mild, moderate, and severe groups was 12.15, 12.65, and 14, respectively. The association between the PREPS score and the umbilical vein flow tested with ANOVA was found to be not significant, with p=0.43. A non-parametric test, Kruskal-Wallis, was additionally done, which also showed a significant association between PREPS score and umbilical artery PI only.

**Table 2:** Shows association between PREPS score and PI of the umbilical artery by ANOVA PREPS: pandemic-related pregnancy stress scale, PI: pulsatility index, *the result is considered statistically significant at a p-value of less than 0.05.

PREPS category	No. of pregnant women (n)	Umbilical artery PI			
		Mean	Std. Dev	Std. Error	
Mild	7	0.74	0.16	0.06	
Moderate	17	0.93	0.19	0.05	
Severe	2	0.63	0.09	0.07	
ANOVA summary					
Source	Degrees of freedom	Sum of squares	Mean square	F-stat	P-value
Between groups	2	0.30	0.15	4.77	0.02*
Within groups	23	0.72	0.03		
Total	25	1.02			

The measurements for biparietal diameter (BPD), abdominal circumference (AC), estimated fetal weight, and femur length were 8.4±0.5, 29.6±2.4, 2427.9±555.6, and 6.6±0.4, respectively. They were found to be within the normal limits for gestational age, and there was no significant association between fetal growth parameters and the PREPS score.

## Discussion

Fetal growth and development are primarily dependent on placental functions. Thus, the placenta is considered to be a critical metabolic organ during pregnancy. As the pregnancy advances, the functional maturity of the placenta plays a major role in maintaining a continued uteroplacental-fetal interaction throughout pregnancy [18]. Nutritional excess or deprivation, advanced maternal age, multiple pregnancies, and stress to the fetus with temperature and high altitude in the sheep model unequivocally proved that the development of normal placental vasculature alone supports normal fetal growth [19]. Restoration of adequate placental blood flow proved to be the prime management strategy for the compromised pregnancy in an overfed, heat-stressed animal model [20].

Abnormal uterine artery Doppler predicted an increased risk of pre-eclampsia and fetal growth retardation [21]. Uterine and umbilical artery blood flow measured by Doppler indirectly denotes the status of fetal and placental blood circulation, respectively [22]. The umbilical venous flow showed a sevenfold increase with the advancement of gestation from 20 to 36 weeks in a normal pregnancy [23]. Reduced umbilical venous blood flow was a predictive marker of growth retardation in fetuses [20]. Quantification of flow characteristics could be done by peak or mean flow velocity, resistance index, and pulsatility index of a pulse wave Doppler [24].

The uterine artery and the umbilical artery PI together were considered surrogate measures of uteroplacental blood flow, which in turn predicted the fetal outcome resulting from the placental vascular impedance [4,13,17,21,22,24]. The normal mean uterine and umbilical artery PIs in the third trimester were 0.98 and 0.95, respectively [25,26]. Contrasting with these values, the mean PI of the uterine and umbilical arteries in this study was found to be lower but within the normal range. This showed the probable occurrence of a compromise in arterial flow with increased stress.

Maternal BMI and PI of the umbilical artery exhibited a positive correlation [4]. Multitudes of tools were used for assessing anxiety, mental well-being, and stress during the perinatal period [10,12,27]. Maternal anxiety was found to cause resistance to uterine artery flow with a notch in the Doppler waveform. Such symptomatic maternal anxiety resulted in a low-birth-weight fetus [13]. Pregnancy-related stress is a predictor of preterm birth [1]. The resistance to placental blood flow mediated by the hypothalamic-pituitary-adrenal axis response to high-level maternal stress was proposed as the probable reason for the increase in preterm deliveries [16]. A statistically significant association between maternal anxiety, mental distress, psychiatric disorder, and Doppler velocimetry of umbilical and uterine arteries was not clearly established [28,29]. Contrary to this, advancement in gestational age and high levels of maternal anxiety were observed to affect the umbilical artery PI, thereby indirectly affecting fetal growth [30].

Psychological worry about their health, relationship, the fetus's health, and having at least one infected family member were considered predictors for low feto-maternal well-being during COVID-19 [10]. Psychological stress symptomatology and COVID-19-related health worries increased in incidence among pregnant women during the pandemic [30]. COVID-19 caused phobia in the anxiety dimension and insomnia in the depression dimension, which were found more among pandemic pregnant women compared to non-pandemic pregnant women [11,27]. The younger chronological age and primigravid status of the pregnancy, which were similar to the profile of pregnant women studied, were found to produce more psychological symptomatology due to COVID-19 [12,27].

The present study was done during the declining phase of the COVID-19 wave in the central Indian region. This explained the negative COVID-19 infection history during the gestational period for the majority of the participants. Missing their antenatal checkup had been reported in pregnant women due to the pandemic in a previous study [12]. A similar observation in the present study needs to be viewed as significant considering the declining phase of the pandemic situation during the study phase. The exploration into the impact of COVID-19 on the livelihood of the participants in this study showed a loss of income and employment for the majority of them. This could be an added stressor observed in these antenatal women.

A very specific questionnaire tool called the pandemic-related pregnancy scale (PREPS) categorized the psychological stress of pregnant women caused by the recent COVID-19 pandemic for the first time in the central India region [15]. Despite the difference in stress tools used in the Mumbai study [12] and the present study, the finding of moderately stressed antenatal women was similar and the highest in percentage. Maternal BMI, anxiety, and stress strongly influenced the PI of the umbilical and uterine arteries [4,13,29,30]. The statistically significant association between umbilical artery PI and PREPS Score in this study adds strength to the hypothesis that maternal stress affects fetal blood flow (Table 2 and Figure 2).

The present study tool focused on delineating the dimensions of psychological stress as preparedness stress and perinatal infection stress, in contrast to the phobia, anxiety, and depression dimensions of the perceived stress scale [11]. The observation that all the category women showed an upper limit in the positive affirmation dimension of the PREPS scale was another positive outcome of the study. The positive affirmation dimension score balanced the scores of the other two dimensions for many of the participants. The pregnant women were probably able to successfully cope with the difficulties, like loss of household income and employment due to the COVID-19 pandemic, by focusing on positivity [15]. Early cognitive therapy and building resilience with a positive outlook on life and pregnancy hold therapeutic value as a management strategy for these antenatal women [15,30].

Study limitations

The small sample size was an analytical limiting factor. The declining phase of the COVID-19 pandemic wherein this study was done was a logistical limiting factor. The profile of the study sample, with a negative history of being infected with COVID-19 during pregnancy, limited the values of the study parameters. If done, the cerebral blood flow recording would have thrown light on the cerebro-placental ratio and the association between maternal stress and fetal outcome.

Future scope

The impact of positive appraisal on pregnancy, especially in challenging life situations, could be explored further.

## Conclusions

The study concludes that COVID-19 has caused stress for antenatal women by way of job loss and economic loss. Seventeen out of twenty-six women studied were moderately stressed. Among the psychological stress dimensions, perinatal infection stress was more prevalent among the women in comparison to preparedness stress. All the women showed a higher limit of positive affirmation dimension on the PREPS scale. The effect of this psychological stress on pregnancy was measured through the pulsatility index of the uterine and umbilical arteries. Even though no significant association between the uterine artery PI and umbilical venous flow PI with PREPS score was found, the umbilical artery PI showed a statistically significant association with the PREPS score. This finding warrants the need for a robust screening of psychological stress in the antenatal period with early intervention and management by cognitive therapy to reduce the adverse impact of placental blood flow and fetal outcomes.
